# Supplementary material for: Signal-Space Projection Suppresses the tACS Artifact in EEG Recordings
Source: Front Hum Neurosci. 2020 Dec 18;14:536070. doi: 10.3389/fnhum.2020.536070 (PMC7775555; doi:10.3389/fnhum.2020.536070)
Supplement: Supplementary file 1 [file Data_Sheet_1.pdf]

Signal-space projection suppresses the tACS artifact in EEG recordings

S11. Supplemental Material and Methods

S11.1. Phantom head construction

The phantom head consisted of two spherical polycarbonate shells with diameters of 120 and 140 mm, with a shell thickness of 2 mm. Similar material was used by Höfner et al. (2011) when studying the ohmic currents produced by a dipolar current source within a spherical object. Both shells consisted of two equally sized hemispheres, which we fixed together with cyanoacrylate (Loctite Precision Super Glue, Henkel AG & Co.KGaA, Düsseldorf, Germany). The two shells were then attached to each other to form a concentric, three-layer structure (Fig. 1).

The smaller sphere was supported from the bottom by four nylon screws that were tightened to holes drilled to the bottom of the larger shell. The largest screw (6 mm wide) was fastened to the south pole of the sphere and the three smaller ones (3 mm wide) were equally spaced being 30 mm away from the South Pole. The smaller sphere was glued to the screws with the cyanoacrylate. The same glue was also used to strengthen the attachment between the screws and the outer spherical shell.

We used 1-mm-thick and 25-mm-long copper-wire segments as electrodes. We attached 22 electrodes on approximate positions of the 10–20 EEG system, on the upper hemisphere of the outer spherical shell so that a 1-mm segment of each electrode wire protruded into the outer sphere (see Fig. 1D). The electrodes were inserted and glued to 2-mm-wide holes with a two-component epoxy glue (Plus Schnellfest, UHU GmbH & CO. KG, Bühl, Germany).

The phantom had a dipolar current element to represent a neuronal current source. The dipole was constructed out of paired cable endings (0.2-mm-thick enameled copper wire) that protruded into the inner shell via two 1-mm holes. The dipolar current source was created by opening a 10-mm-long segment of the paired cable and scratching off the insulating coating from the tip of both wire endings (approx. 1-mm segments). The structure was toughened and fastened to the holes with the epoxy glue.

The dipole was placed on the left hemisphere, on the line between P3 and T5, 10 mm from P3 towards T5, inside the skull (10 mm from the outer surface of the "brain"). Both ends of the dipole were tangential with respect to the skull so that they had an anterior–posterior orientation. After the structure of the phantom was finished, all the seams were sealed with waterproof glue (Casco LiquiSole, Sika Finland AB, Espoo, Finland).

To mimic the conductive properties of the human skull, we drilled 2000 holes with diameter of 1

mm through the inner shell. Via this procedure, the inner shell had a conductivity that is roughly 1/30th of the outer sphere. The holes were randomly and approximately evenly distributed over the spherical surface. Finally, the whole concentric structure was filled with NaCl solution through a 4-mm wide hole on the top of the phantom head using a syringe with a 1.7-mm-thick metal needle. After filling the phantom head, this hole was sealed with a rubber cap. The NaCl solution consisted of 3 g of NaCl mixed with one liter of demineralized water resulting in a conductivity of 0.57 S/m in the brain and the scalp.

Because the current tends to travel the shortest path through the insulating skull and because the well-conducting scalp spreads current components coming through each hole widely across the scalp, we can approximate that for the purpose of EEG the constructed skull appears to have a constant conductivity. This conductivity is determined by the ratio between the total area of the holes and the surface area of the smaller spherical shell. Thus the conductivities of the three layers were 0.57 S/m, 0.019 S/m, and 0.57 S/m for the “brain,” “skull”, and “scalp”. The true conductivities of these tissues are still not well known, but the values here fall well within the variation of most common tissues (Gabriel et al., 2009). The ratio between conductivity of the skull and the brain, which is more important in EEG than the absolute conductivity values, corresponded, e.g., with the findings of Gonçalve et al. (2003) and Lai et al. (2005).

S11.2. SSP details

Let us consider an EEG dataset Y_{tACS} that has been measured during tACS. The measured

EEG dataset consist of the following components:

$Y_{tACS} = GS_{tACS} + A + N$, (SE1)

where S_{tACS}, A, N represent the brain activity during stimulation, tACS-artifacts, and noise signals, respectively. G is the so-called gain or lead-field matrix whose term G_{ij} describes the sensitivity of EEG channel i to neural source j.

If we can assume that the noise and brain signals do not average well across the tACS periods, we can estimate the tACS-artifact topographies from the averaged EEG and project out these topographies using SSP. We write the averaged data in terms of singular-value decomposition:

$Y_{ave} = USV^{T}$, (SE2)

where the column vectors of U, u_k, give all the spatial patterns needed to fully explain the averaged data, and S is a diagonal matrix whose diagonal terms σ_k are the singular values that describe how

much of the total data variance of the averaged data is explained by each spatial pattern u_k. The column vectors of V are the right singular vectors, which are not needed for SSP.

We suppress the tACS artifact by projecting out the K most prominent (based on

their data variance) spatial patterns of Y_{ave} from Y_{tACS} with an operator

$P = I - [u_1, u_2, \ldots, u_K][u_1, u_2, \ldots, u_K]^T$, (SE3)

which approximately fulfills

$PA \approx 0$. (SE4)

This operation removes all the information that can be explained with the spatial patterns u_k, k=1,2,…,K. If the spatial pattern of the artifact does not change over time, K=1 is sufficient for removing the tACS artifact. Otherwise, more components need to be removed. After SSP, the data become:

$PY_{tACS} = PGS_{tACS} + PA + PN = PGS_{tACS} + PN$ (SE5).

Notice, that also the brain and noise signals will be modified to some extent. This can be taken into account with source-informed reconstruction (SIR) (Mutanen et al. 2016) by multiplying the data with a reconstruction operator R. Thus, the cleaned data can be written:

$Y_{clean} = RPY_{tACS} = RPGS_{tACS} + RPN$, (SE6)

where $R = G(PG)^\dagger$, $(\blacksquare)^\dagger$standing for the pseudoinverse.

Even after SIR, SSP attenuates the signals in different channels to some extent. However, analyzing entrainment is still possible because we are usually more interested in the relative changes of oscillatory activity compared to baseline. Since we know exactly the linear operation involved in the SSP cleaning, we can take this information into account when comparing the tACS–EEG signal to the baseline EEG.

Let us consider that we have also performed a baseline measurement that quantifies

spontaneous oscillatory activity. The baseline data Y_{base} can be written as:

$Y_{base} = GS_{base} + N$, (SE7)

where S_{base} contains neural activity during the baseline measurement.

If we now examine the expressions for cleaned and baseline data (Eqs. (SE6) and (SE7)), we see that to demonstrate entrainment, we cannot directly compare the cleaned tACS data to the baseline data. This is because instead of the original gain matrix G, we have the RPG term describing the

sensitivity of each cleaned EEG channel to different neural sources.

To compensate for the distortion introduced by the transform RP, we can apply the same transformation to our baseline data:

$RPY_{base} = RPGS_{base} + RPN.$ (SE8)

If we now observe a change from RPY_{base} to Y_{clean}, we know it is likely to result from a difference in brain activity. This is an advantage of SSP because it allows to take into account the possible overcorrection when comparing the cleaned and the baseline data.

Pseudocode:

1. load data of interest into variable **dataOrig**
2. average **dataOrig** across the tACS cycles and save to **dataTacsAve**
3. form the singular value decomposition (SVD) of **dataTacsAve:**

   [**U, S, V**] = **SVD** (**dataTacsAve**)
4. form the SSP operator **P** in terms of the **K** largest singular directions:

   P = **identityMatrix** - $\mathbf{U}_{1:K}$ * **transpose** ($\mathbf{U}_{1:K}$)
5. load the lead-field matrix of the data into variable **leadField**
6. remove the artifact topographies from **dataOrig** and **leadField**:

   dataAfterSSP = **P** * **dataOrig**

   leadFieldAfterSSP = **P** * **leadField**
7. compute the artifact-free minimum norm estimates **MNE** for the cortical activity:

   MNE = **pseudoinverse** (**leadFieldAfterSSP**) * **dataAfterSSP**
8. project the artifact-suppressed minimum norm estimates back to sensor space:

   dataClean = **leadField** * **MNE**

S11.3. Human

S11.3.1. Paradigm of the human experiment

In an electrically shielded, dimly lit room, the subject (male, 26 years, right-handed) was seated in a recliner in front of a computer screen. EEG was recorded during three blocks of the mental rotation task. The task was to judge whether the two presented stimuli are identical although rotated with respect to each other or whether they are different (Ganis and Kievit 2015; Kasten et al. 2018). Each block consisted of 56 stimuli presented for maximally 7 s each, with an inter-stimulus interval of 3 s. Overall, one block took approximately 9 minutes. The three blocks were recorded without tACS, with weak tACS (0.5 mA) and strong tACS (1 mA).

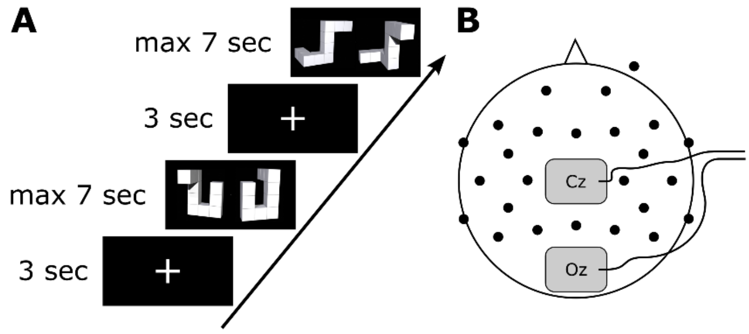

Figure S1. Setup of the human experiment. A: Experimental paradigm showing one trial of the mental rotation experiment. B: Setup of the EEG and stimulation electrodes. Black dots depict EEG electrode positions, grey rectangles stimulation electrode positions.

S11.3.2. Amplitude attenuation after SSP in human data

Comparing artifact-free EEG before and after SSP indicates a slight amplitude reduction regardless of frequency (Fig. S2). The signal amplitude is attenuated to a similar extent at all frequencies because SSP is a spatial filter. Mutanen et al. (2016) showed that SIR can well restore the original neuronally generated topographies but cannot perfectly compensate for the SSP-introduced attenuations in the signal amplitude. Channels with higher pre-SSP amplitudes also showed a stronger absolute amplitude reduction after SSP (Fig. S2, right).

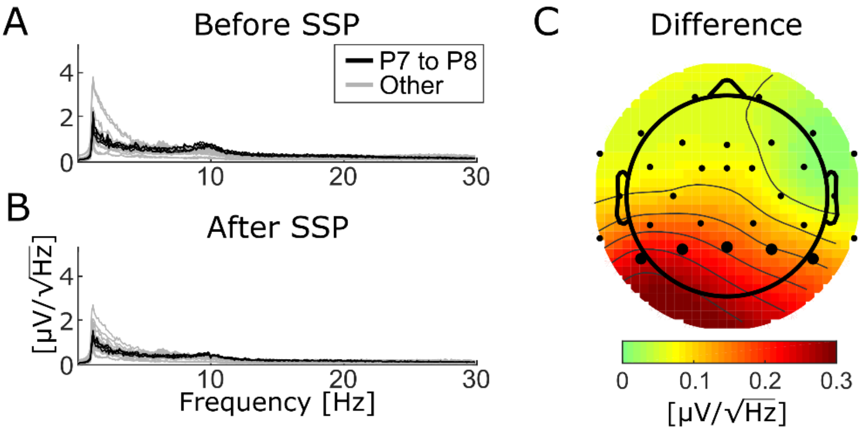

Figure S2: Amplitude attenuation after SSP. Comparison of the tACS-free EEG before (A) and after (B) the SSP algorithm was applied. The alpha peak at 10 Hz is distinguishable before and after but reduced after SSP. Keep in mind that these data represent EEG from a mental rotation task, during which alpha is generally expected to be low. The topography (C) shows the difference in the 8–12-Hz range from before to after SSP correction. Note that there are no occipital channels (O2/O1), and therefore difference values at occipital positions are extrapolated from neighboring electrodes.

S11.3.3. Correlations of topographies after SSP

We correlated the topographies in all possible combinations and revealed highly significant correlations for all possible comparisons of tACS conditions, i.e., Baseline, 500 µA, and 1000 µA (Table S1). Since these comparisons are not solely between conditions, but also resemble different time points of measurement, the correlations between the corrected data are hard to compare. This is different in the EEG data from the phantom, where we could replicate the same signal during all tACS-conditions. A correlation of topographies between corrected phantom data during 50 and 150 µV would ideally be 1.0. In human data, correlations between topographies naturally differ to some extend due to other artifacts different physiological processes.

To provide a comparison of the correlation strengths between conditions after correction, we calculated correlations between topographies from odd and even numbered trials (odd/even split). These values are comparable in size with correlation values between conditions (Table 1). Additionally, we provide the correlation between baseline topographies before versus after SSP which also lies within the same range ($r = 0.85$).

The similarity between the topographies after SSP demonstrate that SSP can successfully recover subtle changes in alpha power on minor scales.

Table S1: Correlations between topographies in different tACS conditions and correlations between odd/even splits within tACS conditions as a control. *p*-values were Bonferroni-corrected for multiple comparisons. The different conditions are: EEG data without tACS artifacts after application of SSP ("SSP Baseline"), data from EEG during 500 µA and 1000 µA tACS and the baseline data before the SSP algorithm was applied ("Original Baseline")

**Experimental Conditions**		**rho**	*p*
SSP Baseline	500 µA	0.73	< .001
SSP Baseline	1000 µA	0.53	.009
500 µA	1000 µA	0.70	< .001
**Control Correlations**			
Baseline odd	Baseline even	0.77	< .001
500 µA odd	500 µA even	0.45	.001
1000 µA odd	1000 µA even	0.68	< .001
Original Baseline	SSP Baseline	0.85	< .001

S11.4. References (Supplemental)

Gonçalve, S., De Munck, J. C., Verbunt, J., Bijma, F., Heethaar, R. M., & Lopes da Silva, F. (2003). In vivo measurement of the brain and skull resistivities using an EIT-based method and realistic models for the head. *IEEE Transactions on Biomedical Engineering*, 50(6), 754–767.

Gabriel, C., Peyman, A., & Grant, E. H. (2009). Electrical conductivity of tissue at frequencies below 1 MHz. Physics in Medicine and Biology, 54(16), 4863.

Ganis, G., & Kievit, R. (2015). A New Set of Three-Dimensional Shapes for Investigating Mental Rotation Processes: Validation Data and Stimulus Set. *Jounal of Open Psychololgy Data*, 3(1), p.e3.

Höfner, N., Albrecht, H.H., Cassará, A.M., Curio, G., Hartwig, S., Haueisen, J., Hilschenz, I., Körber, R., Martens, S., Scheer, H.J., Voigt, J., Trahms, L., & Burghoff, M. (2011). Are brain currents detectable by means of low-field NMR? A phantom study. *Magnetic Resonance Imaging*, 29(10), 1365–1373.

Kasten, F.H., Maess, B., & Herrmann, C.S. (2018). Facilitated Event-Related Power-Modulations during Transcranial Alternating Current Stimulation (tACS) Revealed by Concurrent tACS-MEG. *Eneuro*, 5:ENEURO.0069-18.2018.

Lai, Y., Van Drongelen, W., Ding, L., Hecox, K. E., Towle, V. L., Frim, D. M., & He, B. (2005). Estimation of in vivo human brain-to-skull conductivity ratio from simultaneous extra-and intra-cranial electrical potential recordings. *Clinical Neurophysiology*, 116:456-465.

Mutanen, T. P., Kukkonen, M., Nieminen, J. O., Stenroos, M., Sarvas, J., & Ilmoniemi, R. J. (2016). Recovering TMS-evoked EEG responses masked by muscle artifacts. *NeuroImage*, 139:157–166.
